# Benign Subcutaneous Nodules and Hypereosinophilic Syndrome: A Rare Presentation of an Uncommon Entity

**DOI:** 10.1155/2019/2387832

**Published:** 2019-11-18

**Authors:** Rimesh Pal, Uday Yanamandra, Prasanna Kumar, Nirmalya Banerjee

**Affiliations:** ^1^Post Graduate Institute of Medical Education and Research, Chandigarh, India; ^2^Army Research and Referral Hospital, New Delhi, India

## Abstract

A 40-year-old gentleman presented with a history of multiple swellings involving his face, scalp, left axilla, back, and right thigh for the past 8 years. For the last 6 months, he developed intermittent low-grade fever, anorexia, weight loss, and gradually worsening breathlessness. On evaluation, the patient was found to have abnormally elevated absolute eosinophil count. Workup for the etiology of eosinophilia was unrewarding. All investigations related to an underlying myeloproliferative disorder were negative. Hence, a clinical possibility of angiolymphoid hyperplasia with eosinophilia (ALHE) was kept which was confirmed on histopathology. In the absence of other causes of hypereosinophilia, a clinical diagnosis of “associated hypereosinophilic syndrome” secondary to ALHE was made. He was managed with oral corticosteroids. The absolute eosinophil count reduced markedly, while the swellings showed a more gradual response, shrinking in size by about 50% following two months of therapy. The index case thus highlights a rather unusual presentation of ALHE.

## 1. Introduction

Hypereosinophilic syndromes (HESs) are a heterogeneous group of disorders characterized by a peripheral blood eosinophil count of 1.5 × 10^9^/L or higher with evidence of end-organ damage attributable to eosinophilia and unexplained by other pathological conditions. HES could be idiopathic or associated with a myriad of underlying conditions. Accordingly, HES can be classified into six subtypes: myeloproliferative HES, lymphocytic variant HES, overlap HES, associated HES, familial HES, and idiopathic HES [1]. Associated HES is characterized by reactive eosinophilia in the setting of a distinct diagnosis. It accounts for 10–20% of all cases of unexplained HES. Hypereosinophilia tends to resolve with targeted treatment of the underlying cause [2]. Conditions classically leading to associated HES are parasitic, allergic, rheumatological, vasculitic, and neoplastic disorders. Herein, we describe a middle-aged man presenting with hypereosinophilic syndrome, who on evaluation, was found to have angiolymphoid hyperplasia with eosinophilia (ALHE), a benign vascular proliferative disorder of unknown etiology. Hypereosinophilia was attributed to ALHE after ruling out other causes. He was treated with oral corticosteroids, resulting in symptomatic improvement and a reduction in the size of the skin lesions.

## 2. Case Presentation

A 40-year-old gentleman presented to our institute (Postgraduate Institute of Medical Education and Research) with the history of multiple swellings over his body. He had first noticed them 8 years back when a small lump had developed over his left cheek. This was followed by similar swellings over the scalp, left axilla, back, and right thigh. They were slow growing, pruritic, painless, and apart for cosmetic reasons, and they were nonbothersome to the patient. Hence, over the years, he did not seek any medical attention. However, over the past 6 months he started developing intermittent low-grade fever, anorexia, involuntary weight loss, and gradually worsening breathlessness. When probed, he also gave a history of numbness in his bilateral feet. He denied any history of addictions or over-the-counter drug intake.

**Figure 1:**
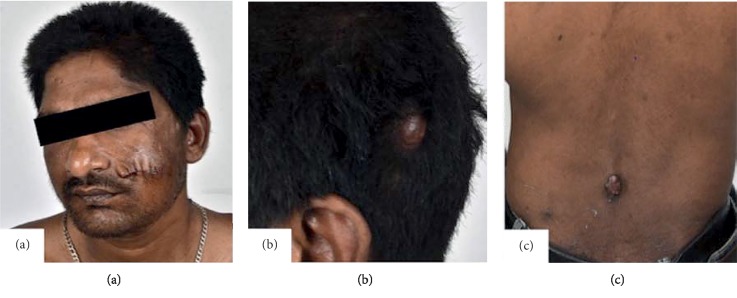
Figure illustrating the swelling over the left side of the face after excision with suture marks (a), with nodular swellings over the scalp (b) and over the lower back (c).

On examination, he was tachypnoeic (respiratory rate: 24/min). There were multiple swellings over the aforementioned regions (Figure 1). Most of them were small (measuring about 2 × 2 cm), brownish in color, and had a soft, fleshy feel. There was no hepatosplenomegaly or clinically significant lymphadenopathy. On auscultation, there was diffuse polyphonic wheeze. He had graded loss of pain, touch, and temperature sensations from the toes to the shin. Bilateral ankle jerks were not elicitable.

**Figure 2:**
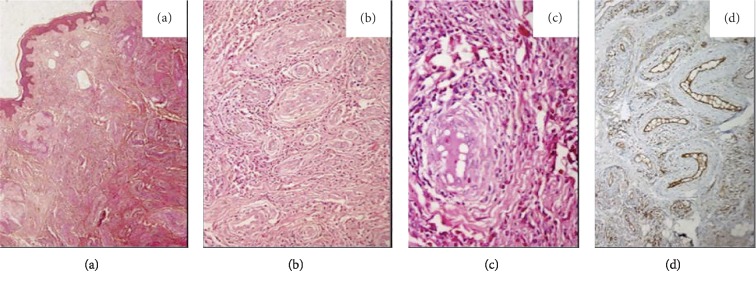
Photomicrograph showing a circumscribed lesion located in the dermis, composed of variably sized vascular spaces with cellular infiltrate (a) (H&E stain, 4x). The lesion is showing vascular spaces with plump endothelial cells and interstitial increase in eosinophil, lymphocyte, and plasma cells (b) (H&E stain 10x) and (c) (H&E stain, 20x). Immunohistochemistry showing endothelial cells positive for CD31 (d).

Investigations revealed anemia (hemoglobin = 10.8 gm/dL) and persistent leukocytosis with an eosinophilic predominance. Absolute eosinophil count ranged from 36,000/*μ*L to 44,000/*μ*L. Peripheral blood smear, however, did not reveal any blasts or any abnormal cell lineages. Biochemical investigations were essentially normal. Renal function test was normal (serum creatinine: 0.8 mg/dl; range: 0.6–1.2). Urinalysis did not reveal any active sediment. Stool examination did not show any ova or parasites. His HIV serology and filarial serology were negative. Anti-nuclear antibody (by ELISA) and anti-neutrophil cytoplasmic antibody (by indirect immunofluorescence) were also negative. Chest radiograph and contrast-enhanced computed tomography of the neck, chest, and abdomen were performed which did not reveal any mediastinal or abdominal lymphadenopathy neither any mass lesion in the thorax or abdomen. His serum angiotensin-converting enzyme level was normal. Workup for allergic bronchopulmonary aspergillosis was negative. However, his serum total IgE level was elevated (36 kU/l (normal <1.58 kU/l)). Considering the possibility of an underlying myeloproliferative disorder, a bone marrow examination was performed which showed increased eosinophil and their precursors (16%) with normal blast cell count (1%). There were no aberrant T-cell clones. Karyotype was normal, and fluorescence in situ hybridization (FISH) studies for *BCR-ABL, JAK2, PDGFRA, PDGFRB*, and *FGFR1* were all negative. Histopathology of the excised lump on the left cheek showed a circumscribed lesion located in the dermis, composed of variably sized vascular spaces lined by plump endothelial cells and interstitial increase in eosinophil, lymphocyte, and plasma cells. The endothelial cells were positive for CD31, suggestive of ALHE (Figure 2). In the absence of other causes of hypereosinophilia, a diagnosis of associated HES secondary to ALHE was made. Two-dimensional echocardiography ruled out cardiac involvement. Spirometry showed reversible airway obstruction, while nerve conduction study was suggestive of axonal sensorimotor polyneuropathy in bilateral lower limbs.

The patient was managed with oral corticosteroids at an initial dose of 1 mg/kg of body weight. He was also managed with inhaled bronchodilators for symptomatic relief. There was marked improvement in his breathlessness, and absolute eosinophil count reduced to 12,000/*μ*L after 2 days of steroids. The swellings over his body showed a more gradual response, and when followed up at 2 months, they had reduced in size by about 50%. The last recorded absolute eosinophil count was 800/*μ*L. His serum total IgE level had come down to 2.2 kU/l. Further plan is to gradually taper the dose of oral corticosteroids over a period of 4 months.

## 3. Discussion

ALHE is a rare, benign vascular proliferative disorder of no definite etiology. The first case of ALHE was reported by Wells and Whimster way back in 1969 [3]. It was earlier believed to be predominantly a disease of young to middle-aged women [4]. However, a recent systematic review of over 400 studies showed that the mean age at presentation was 37.6 years with no sex predominance. The lesions tend to have a predilection for the head and neck region (the ear and the periauricular area being the most common) although they can occur in any part of the body [5]. Lesions are solitary or multiple and are pink to red-brown papules or nodules. Pruritus is the most common complaint although pain and spontaneous bleeding have also been reported. The closest differential diagnosis of ALHE is Kimura disease, a benign chronic inflammatory disease of unknown etiology. Kimura disease is more common in Asian men, characterized by deep-seated subcutaneous nodules and usually associated with lymphadenopathy, renal and salivary gland involvement, peripheral eosinophilia, and elevated serum IgE. ALHE, on the other hand, is characterized by smaller and more superficial swellings. Regional lymphadenopathy is uncommon, and usually, serum IgE and absolute eosinophil count are not increased [6]. Histopathology helps in confirming the diagnosis.

The index case is extremely unique in being associated with hypereosinophilic syndrome (HES). As has already been talked of, HES occurring in the setting of a distinct diagnosis is referred to as associated HES [1]. Common clinical conditions associated with HES include helminthic infections, sarcoidosis, IgG4-related disease, primary immunodeficiency syndromes, and drug hypersensitivity. ALHE as a cause of associated HES is an extreme rarity. Olsen and Helwig in the series of 116 patients of ALHE found that only 20% of the cases had peripheral blood eosinophilia ranging from 6% to 34% [4]. Elevated interleukin-5 (IL-5) levels have been reported in patients with AHLE, probably contributing to its pathogenesis as well. IL-5 in turn has been implicated in eosinophil proliferation and differentiation. Although IL-5 levels were not measured in our patient, it could be that very high IL-5 levels might have led to a hypereosinophilic state in the index case.

As per as management is concerned, hitherto, no evidence-based consensus exists. Both medical and surgical therapies have been tried in patients with ALHE, with variable success. Surgical therapy includes excision of the lesions but is fraught with recurrence, scars (obviating its use in cosmetic sites and over the joints), and difficulty in multiple sites. Mohs microsurgery, pulsed dye laser, carbon dioxide laser, cryosurgery, electrosurgery, and 5-aminolevulinic acid photodynamic therapies have been tried in ALHE with variable success [5, 7–9]. The most effective of the medical therapy includes intralesional steroids and oral propranolol therapy [10, 11]. Oral propranolol therapy has also been previously successfully used in infantile hemangiomas [11]. Systemic corticosteroids; mepolizumab, an anti-IL-5 antibody; and isotretinoin are the other reported systemic therapies [12, 13]. Various topical agents tried in ALHE include intralesional IFN2α, topical tacrolimus, imiquimod, and isotretinoin, of which topical timolol is reported to have some success [14–16]. We managed our patient with systemic steroids owing to costs and logistics involved with the other agents in the real-world settings with good results.

In conclusion, we have described a unique case of ALHE, presenting with multiple benign subcutaneous swellings along with HES. Thus, although rare, ALHE should be considered as a cause of associated HES and should be included in the differential diagnoses after having excluded more common secondary causes of HES.
